# Hook Wire Placement Facilitates Laparoscopic Excision of Endophytic Renal Tumor in Partial Nephrectomy

**DOI:** 10.1089/cren.2018.0054

**Published:** 2018-10-01

**Authors:** Qusay Mandoorah, Francois Rozet, Fabio Muttin, Eric Barret, Rafael Sanchez-Salas, Xavier Cathelineau

**Affiliations:** Department of Urology, Institut Mutualiste Montsouris, Paris, France.

**Keywords:** partial nephrectomy, hook wire, endophytic renal tumor

## Abstract

***Introduction:*** Endophytic renal tumors that are completely intraparenchymal pose several challenges to surgeons, including in intraoperative tumor identification. Image-guided hook wires, which are now used in surgery, particularly in spinal surgery, thoracoscopic surgery, and breast surgery, allow for the precise localization of tumor sites. The hook wire facilitated the localization of the lesion and avoided cutting into the lesion directly.

***Case Presentation:*** A 55-year-old woman was referred to our hospital due to an incidentally discovered 16-mm intracortical right renal mass in the anterior medial position. A renal biopsy was performed, which confirmed renal cell carcinoma. A hook wire was placed in the tumor by an interventional radiologist under CT guidance. This was done before performing the partial nephrectomy on the same day. The hook wire was found intraoperatively, and the renal artery was clamped. The renal capsule was resected using scissors under warm ischemia (25 minutes). Histopathology confirmed clear-cell renal carcinoma with negative surgical margins, Classification TNM 2017: pT1a Nx.

***Conclusion:*** Use of a hook wire is an alternative method for localizing endophytic lesions in partial nephrectomy.

## Introduction

Endophytic renal tumors that are completely intraparenchymal pose several challenges to surgeons, including an intraoperative tumor identification.^1^ Image-guided hook wires, which are now used in surgery, particularly in spinal surgery, thoracoscopic surgery, and breast surgery, allow for the precise localization of tumor sites.^2,3^ We report the case of a woman undergoing laparoscopic partial nephrectomy in which a hook wire was used. The hook wire facilitated the localization of the lesion and avoided cutting into the lesion directly. We found one case report involving embolization coil placement.^4^

## Case Presentation

**FIG. 1.**
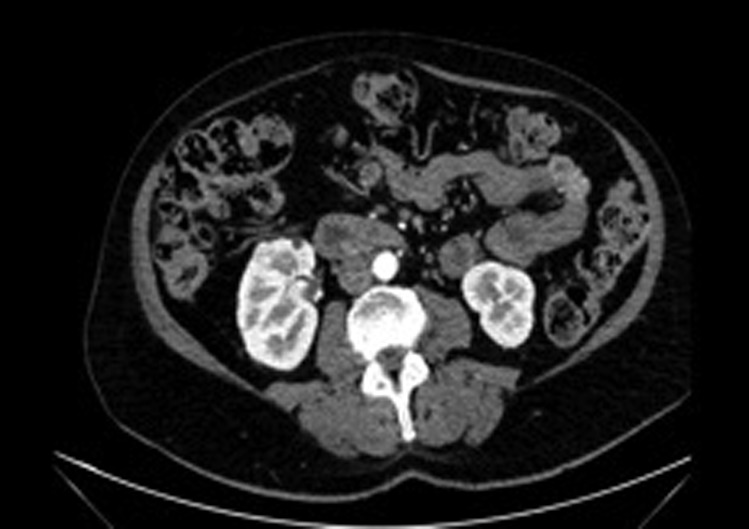
Computed tomographic scan views of the right renal mass.

A 55-year-old woman was referred to our hospital due to an incidentally discovered 16-mm intracortical right renal mass in the anterior medial position (Fig. 1). A renal biopsy was performed, which confirmed renal cell carcinoma.

**FIG. 2.**
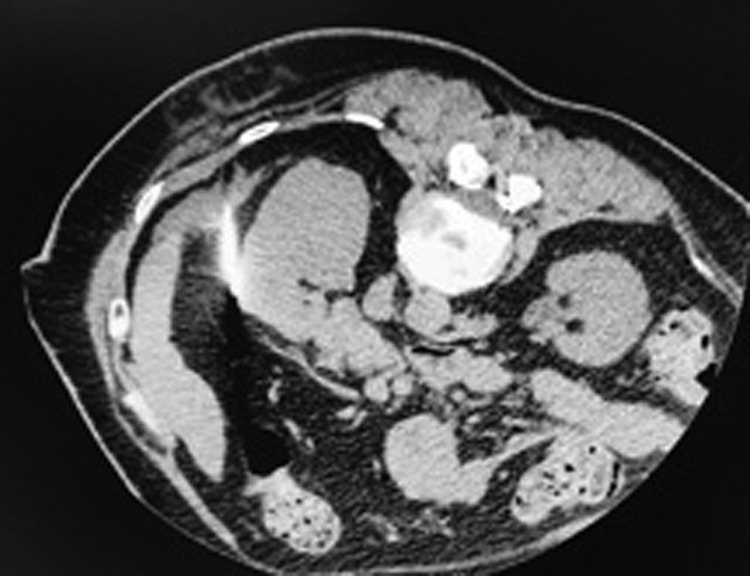
Computed tomographic scan views after the placement of the hook wire.

**FIG. 3.**
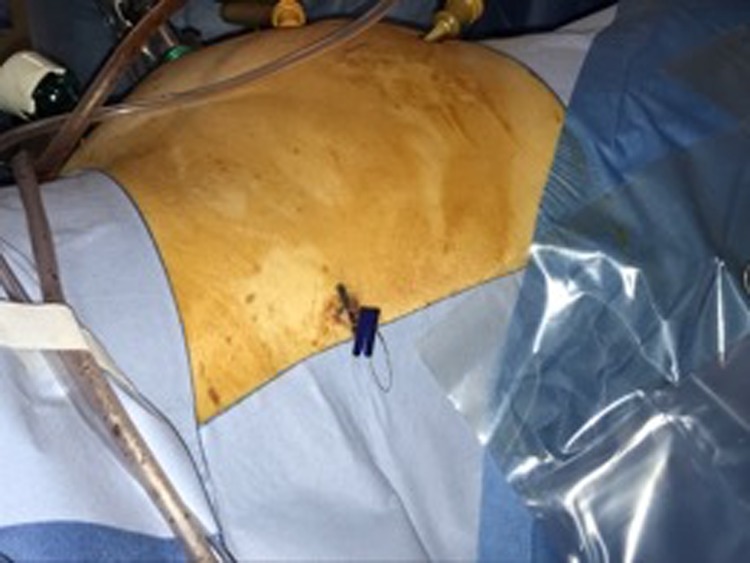
Placement of the hook wire extracorporeal.

A hook wire was placed in the tumor by an interventional radiologist under CT guidance. This was done before performing the partial nephrectomy on the same day using the technique used for nonpalpable breast lesions to facilitate intraoperative localization of the tumor (Figs. 2 and 3).

**FIG. 4.**
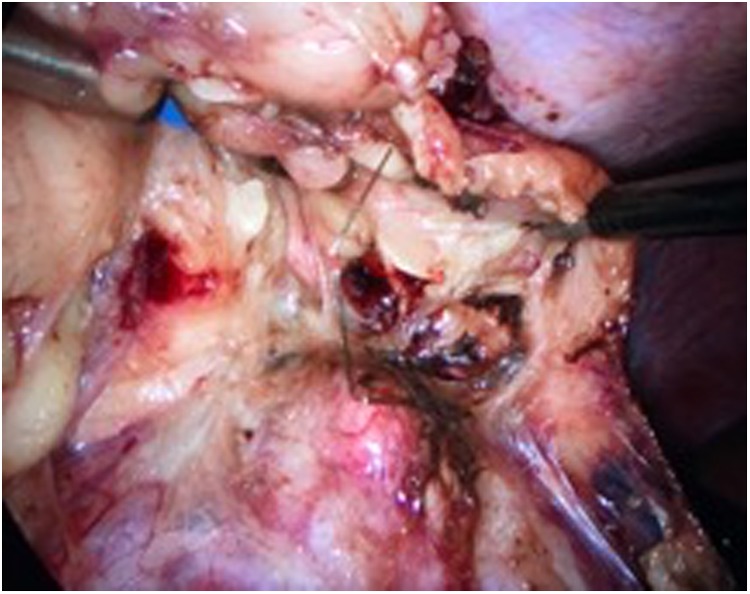
Intraoperative view; the hook wire can be observed.

The patient was placed in a modified left lateral decubitus position. Pneumoperitoneum was established and the trocars were placed. The bowel was mobilized medially and the plane between the anterior Gerota's fascia and the posterior mesocolon was developed. The kidney was mobilized within Gerota's fascia. The hook wire was found, and the renal artery was clamped by the bulldog. The renal capsule was resected using scissors under warm ischemia (25 minutes) (Fig. 4).

Hemostasis was achieved using a combination of cautery, hemostatic agents, and suturing (using sutures preloaded with clips to secure a suture line allows for lateral compression, perpendicular to the renal capsule).

Histopathology confirmed clear-cell renal carcinoma with negative surgical margins, Classification TNM 2017: pT1a Nx.

## Discussion

Laparoscopic partial nephrectomy for endophytic renal masses is technically challenging, as the surgeon does not have intraoperative gross observation of the renal mass and recognition of the mass extension. Image-guided hook wires, which are now used in surgery, particularly in spinal surgery, thoracoscopic surgery, and breast surgery, allow for the precise localization of tumor sites. The advantages of using this technique are intraoperatively easy to identify the needle and short localization procedure time. However, it also has disadvantages including hook wire dislodgement that could occur during patient transport and positioning or surgeon manipulation of the kidney.

Intraoperative ultrasonography is widely utilized for localization. It improves the identification of the renal mass location and facilitates complete tumor excision with negative surgical margins. Real-time three-dimensional (3D) image guidance system by Tilepro™ is reportedly also used. This system (renal tumor and vascular 3D image navigation shown on the surgeon console view through Tilepro) facilitates tumor and vascular identification, precise dissection of both the tumor and the pedicle, and selective clamp techniques. Another technique that facilitates tumor identification is near-infrared fluorescence imaging using indocyanine green dye.^1^ In the absence of these techniques, the hook wire technique is an alternative method for tumor localization and can be combined with intraoperative ultrasonography.

## Conclusion

Use of a hook wire is an alternative method for localizing endophytic lesions in partial nephrectomy.

## Abbreviations Used

3D: three-dimensional
CT: computed tomography
